# Effects of sampling seasons and locations on fish environmental DNA metabarcoding in dam reservoirs

**DOI:** 10.1002/ece3.6279

**Published:** 2020-05-06

**Authors:** Kana Hayami, Masayuki K. Sakata, Takashi Inagawa, Jiro Okitsu, Izumi Katano, Hideyuki Doi, Katsuki Nakai, Hidetaka Ichiyanagi, Ryo O. Gotoh, Masaki Miya, Hirotoshi Sato, Hiroki Yamanaka, Toshifumi Minamoto

**Affiliations:** ^1^ Graduate School of Human Development and Environment Kobe University Kobe Japan; ^2^ OYO Corporation Miharu‐machi Japan; ^3^ Graduate School of Humanities and Sciences Nara Women's University Nara Japan; ^4^ Graduate School of Simulation Studies University of Hyogo Kobe Japan; ^5^ Lake Biwa Museum Kusatsu Japan; ^6^ Water Resources Environment Center Chiyoda‐ku Japan; ^7^ Natural History Museum and Institute Chiba Chiba Japan; ^8^ Graduate School of Human and Environmental Studies Kyoto University Kyoto Japan; ^9^ Faculty of Science and Technology Ryukoku University Otsu Japan; ^10^ Center for Biodiversity Science Ryukoku University Otsu Japan

**Keywords:** environmental DNA, fish, metabarcoding, reservoir

## Abstract

Environmental DNA (eDNA) analysis has seen rapid development in the last decade, as a novel biodiversity monitoring method. Previous studies have evaluated optimal strategies, at several experimental steps of eDNA metabarcoding, for the simultaneous detection of fish species. However, optimal sampling strategies, especially the season and the location of water sampling, have not been evaluated thoroughly. To identify optimal sampling seasons and locations, we performed sampling monthly or at two‐monthly intervals throughout the year in three dam reservoirs. Water samples were collected from 15 and nine locations in the Miharu and Okawa dam reservoirs in Fukushima Prefecture, respectively, and five locations in the Sugo dam reservoir in Hyogo Prefecture, Japan. One liter of water was filtered with glass‐fiber filters, and eDNA was extracted. By performing MiFish metabarcoding, we successfully detected a total of 21, 24, and 22 fish species in Miharu, Okawa, and Sugo reservoirs, respectively. From these results, the eDNA metabarcoding method had a similar level of performance compared to conventional long‐term data. Furthermore, it was found to be effective in evaluating entire fish communities. The number of species detected by eDNA survey peaked in May in Miharu and Okawa reservoirs, and in March and June in Sugo reservoir, which corresponds with the breeding seasons of many of fish species inhabiting the reservoirs. In addition, the number of detected species was significantly higher in shore, compared to offshore samples in the Miharu reservoir, and a similar tendency was found in the other two reservoirs. Based on these results, we can conclude that the efficiency of species detection by eDNA metabarcoding could be maximized by collecting water from shore locations during the breeding seasons of the inhabiting fish. These results will contribute in the determination of sampling seasons and locations for fish fauna survey via eDNA metabarcoding, in the future.

## Graphical abstract

Number of detected species by eDNA metabarcoding for (a) Miharu dam reservoir, (b) Okawa dam reservoir, and (c) Sugo dam reservoir. The number of species detected by eDNA survey peaked in May in Miharu and Okawa reservoirs, and in March and June in Sugo reservoir, which corresponds with the breeding seasons of many of the fish species inhabiting the reservoirs.

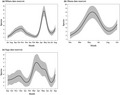

## 1 INTRODUCTION

A novel biodiversity monitoring method called environmental DNA (eDNA) analysis has seen rapid development in the last decade (Ficetola, Miaud, Pompanon, & Taberlet, 2008; Minamoto, Yamanaka, Takahara, Honjo, & Kawabata, 2012; Takahara, Minamoto, Yamanaka, Doi, & Kawabata, 2012). Environmental DNA refers to DNA released by organisms into their environments, for example, in the form of shed cells and other biological material, or decaying matter (Barnes & Turner, 2016). This method is noninvasive, as species can be identified simply by collecting water and investigating DNA information contained in the water, and there are several advantages regarding cost and efficiency when compared with conventional survey methods such as capturing and visual inspection (Darling & Mahon, 2011; Jerde, Mahon, Chadderton, & Lodge, 2011; Olson, Briggler, & Williams, 2012).

Early studies primarily focused on detecting a single or several specific species that inhabit various aquatic environments. Such species‐specific approaches have proven highly successful for detecting individual species from a wide range of taxonomic groups in aquatic environments (Goldberg, Sepulveda, Ray, Baumgardt, & Waits, 2013; Sakata, Maki, Sugiyama, & Minamoto, 2017; Takahara, Minamoto, & Doi, 2013). Although species‐specific approaches are powerful in monitoring the target species, they are not suitable for the assessment of community composition in certain ecosystems, which requires a great deal of effort. Therefore, in addition to the species‐specific approaches, eDNA metabarcoding, in which various kinds of DNA fragments present in the aquatic environments can be simultaneously analyzed, has emerged (Civade et al., 2016; Kelly, Port, Yamahara, & Crowder, 2014; Miya et al., 2015; Thomsen et al., 2012; Valentini et al., 2016). Environmental DNA metabarcoding is a method of collectively amplifying the DNA fragments of a certain taxonomic group, determining their nucleotide sequences using a high‐throughput sequencing (HTS) technology and identifying the species composition by comparing with sequence databases.

In recent years, eDNA metabarcoding is being used increasingly to characterize the species compositions of ecological communities (Bista et al., 2017; Blackman et al., 2017; Grey et al., 2018; Komai, Gotoh, Sado, & Miya, 2019). To assess biodiversity and community composition of organisms using eDNA metabarcoding, effective protocols are required at each step. This includes the following: DNA sampling and collection (Mächler, Deiner, Spahn, & Altermatt, 2015; Sato, Sogo, Doi, & Yamanaka, 2017), choice of markers (Evans et al., 2016; Hänfling et al., 2016), DNA extraction (Deiner, Walser, Mächler, & Altermatt, 2015; Eichmiller, Miller, & Sorensen, 2016), PCR (Doi et al., 2019; Ficetola et al., 2015), bioinformatics analyses, and taxonomic assignment of sequences (Ficetola, Taberlet, & Coissac, 2016).

In particular, the first step of the eDNA survey, which involves collecting water samples, is critically important because sampling efforts are a fundamental aspect of any ecological study or monitoring procedure and thus may deeply affect results and interpretations (Cantera et al., 2019). Previous species‐specific eDNA studies on fish and amphibians that targeted one or multiple species have already reported that the power of the species‐specific detection increased when sampling was performed during the breeding season (Buxton, Groombridge, Zakaria, & Griffiths, 2017; Hinlo, Furlan, Suitor, & Gleeson, 2017; Thomsen & Willerslev, 2015). Other studies have reported clear differences in the spatial distribution of eDNA (Hänfling et al., 2016; Port et al., 2016; Zhang et al., 2020), or compositional differences in accordance with the seasonal variations in the fish community. This phenomenon is similar to the seasonal patterns recovered by conventional surveys (Sigsgaard et al., 2017; Stoeckle, Soboleva, & Charlop‐Powers, 2017; Zou et al., 2020). However, none of these studies examine the temporal and spatial changes in species detection throughout the year at multiple survey sites using an identical method. We therefore emphasize the importance to examine the temporal and spatial changes in species detection throughout the year at multiple survey sites using an identical method.

In this study, we aimed to identify the optimal sampling season and location, to maximize species detection for the characterization of entire fish communities, using eDNA metabarcoding in dam reservoirs of various sizes. To achieve this, we conducted water sampling in three dam reservoirs, in different seasons and at different locations, and examined the data for: (a) difference between conventional and eDNA surveys for species detection, (b) temporal difference throughout the year, and (c) spatial difference between offshore and shore sampling. Based on the results of these analyses, we tried to identify the optimal sampling strategy for eDNA metabarcoding analysis, that is, one that could detect the maximum number of species with the minimum effort.

## 2 METHODS

### 2.1 Study sites

**FIGURE 1:**
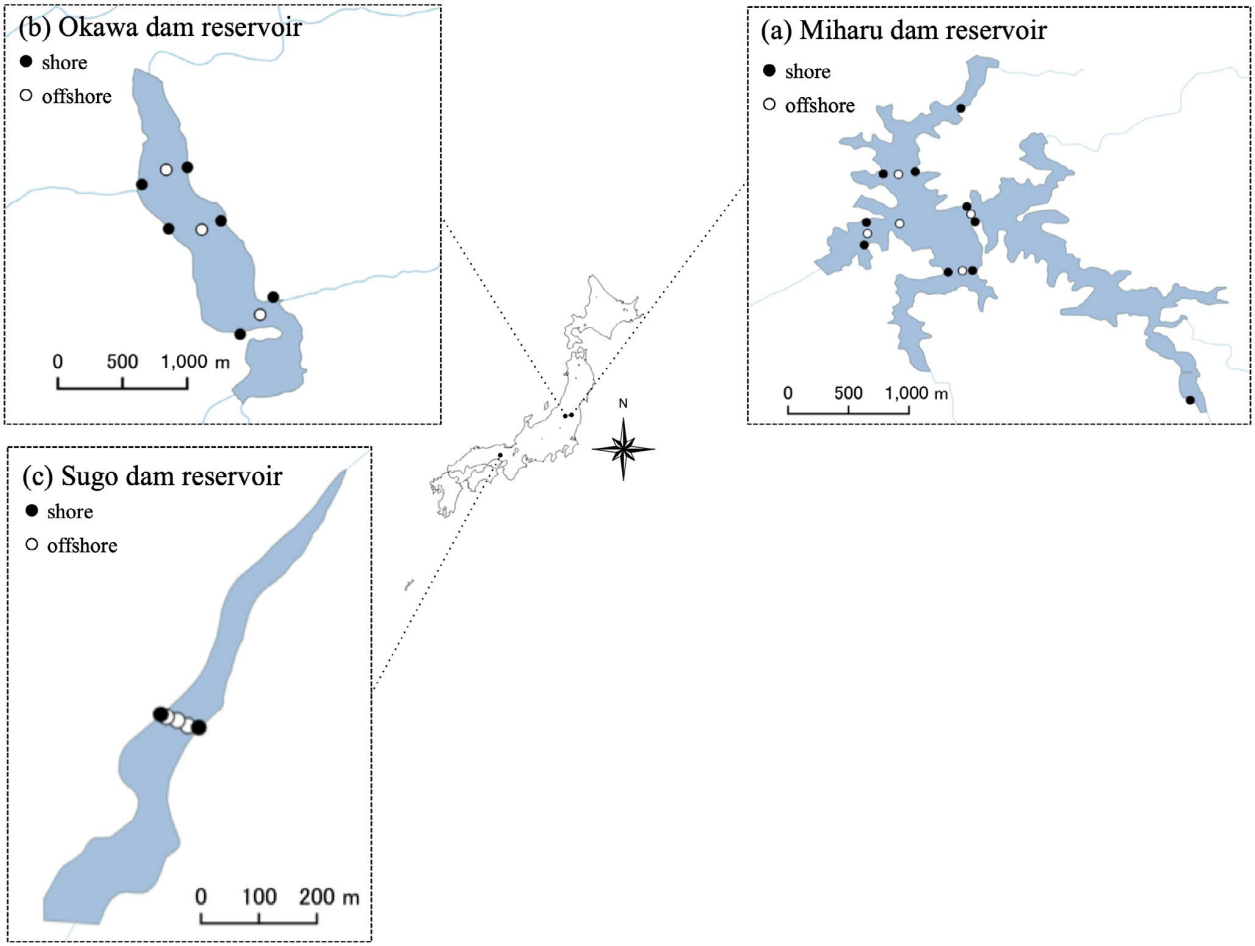
Sampling sites in the three dam reservoirs investigated. (a) Miharu dam reservoir and (b) Okawa dam reservoir in Fukushima Prefecture, and (c) Sugo dam reservoir in Hyogo Prefecture, Japan. Solid and open circles show shore and offshore sites, respectively

We conducted field surveys in three dam reservoirs: Miharu (37º24′14″N, 140º28′29″E) and Okawa (37º20′47″N, 139º54′43″E) dam reservoirs in Fukushima Prefecture, and Sugo dam reservoir in Hyogo Prefecture (35º00′05″N, 134º38′01″E) of Japan (Figure 1; Table 1). In the Miharu dam reservoir, we conducted a total of 14 surveys between July 2015 and August 2016, once every month. The sampling points were set at 15 locations (five offshore and 10 shore sites) within the reservoir (Figure 1a; Appendix S1a). In Okawa dam reservoir, we conducted a total of six surveys, one each in December 2015 and in March, May, July, August, and October 2016, approximately every 2 months, and the sampling points were set at nine locations (three offshores and six shores) within the reservoir (Figure 1b; Appendix S1b). Offshore sites were about 65–95 m and 80–120 m away from the nearest shore sites within the Miharu and Okawa dam reservoirs, respectively. In Sugo dam reservoir, we conducted a total of 12 surveys, once every month from September 2015 to July 2016 and one in September 2016. The sampling points were set at five locations (three offshores and two shores) from the shore to the opposite shore at 15 m intervals within the reservoir (Figure 1c; Appendix S1c). The number of sampling points in each dam reservoir was set according to the surface area of the reservoir (Miharu: 290 ha, Okawa: 190 ha, Sugo: 13 ha as seen in Table 1).

**TABLE 1:** Basic information on the three dam reservoirs that are surveyed. Average depth is effective storage capacity divided by submerged area. Turnover rate is annual inflow divided by reservoir capacity

	Miharu dam	Okawa dam	Sugo dam
Submerged area	2,900,000 m^2^	1,900,000 m^2^	130,000 m^2^
Reservoir capacity	42,800,000 m^3^	57,500,000 m^3^	1,950,000 m^3^
Effective storage capacity	36,000,000 m^3^	44,500,000 m^3^	1,700,000 m^3^
Annual inflow			
2015	169,980,000 m^3^	1,127,660,000 m^3^	13,110,000 m^3^
2016	147,990,000 m^3^	754,340,000 m^3^	10,820,000 m^3^
Average depth	12.4 m	23.4 m	15.0 m
Turnover rate			
2015	4.0 times/year	19.6 times/year	6.7 times/year
2016	3.5 times/year	13.1 times/year	5.5 times/year

### 2.2 eDNA sampling and filtering

At each site, we collected 1 L of surface water using a 1‐L plastic bottle, and benzalkonium chloride was added at a final concentration of 0.01% to suppress DNA degradation (Yamanaka et al., 2017). All samples were collected from a boat and were not replicated at any of the sites. In Sugo dam reservoir, 2 L of ultrapure water was prepared as a field control (field blanks). In the subsequent processing, field blanks were treated the same as field samples. The water samples were transported to the laboratory, and 1 L of each sample was filtered using 47‐mm glass‐fiber filters with nominal pore size of 0.7 μm (GF/F, GE Healthcare), within 24 hr of sampling. A 1 L sample of pure water was also filtered to monitor for contamination at the filtration and the subsequent extraction steps (filtration blanks). The filters were preserved at −25℃ until DNA extraction. All equipment used in water collection and water filtration steps, including polyethylene tanks, filter funnels, and tweezers, were bleached using a diluted commercial bleach solution (>0.1% sodium hypochlorite solution) before use, to prevent contamination. Disposable gloves were used during all procedures to minimize the risk of contamination.

### 2.3 DNA extraction

Extraction of eDNA from the filters was performed as described in a previous study (Uchii, Doi, & Minamoto, 2016) with slight modifications. In brief, each filter was placed in a Salivette tube (Sarstedt) and 440 μl lysis solution, composed of 400 μl of Buffer AL (Qiagen) and 40 μl of Proteinase K (Qiagen), was added to the filters without adding Buffer ATL (Qiagen) according to previous reports (Doi et al., 2017; Fujii et al., 2019; Minamoto, Hayami, Sakata, & Imamura, 2019). The tubes were then incubated at 56°C for 30 min. After incubation, the Salivette^®^ tubes were centrifuged at 3,000 *g* for 3 min to collect the DNA. To increase DNA yield, 300 μl of Tris‐EDTA (TE) buffer was added to the filters and they were re‐centrifuged at 3,000 *g* for 1 min. The collected DNA was purified using the DNeasy Blood & Tissue Kit (Qiagen) according to the manufacturer's protocol. The extracted DNA samples (100 μl) were stored at −25°C until the PCR assay. Subsequently, filtration blanks (32 blanks in total) were treated in the same way as field samples. To avoid contamination, we performed filtration and DNA extraction (room 1), and two‐step PCR (room 2), in two separate rooms. To prevent carryover contamination, no equipment or samples were moved from room 2 to room 1.

### 2.4 Paired‐end library preparation and MiSeq sequencing

A two‐step PCR was used to prepare the libraries for Illumina MiSeq sequencing. As the first step, a fragment of the mitochondrial 12S rRNA gene was amplified using the MiFish‐U primers targeting teleost fish (Miya et al., 2015), which were designed to contain Illumina sequencing primer regions and six random bases (N) (forward: 5′‐ACACTCTTTCCCTACACGACGCTCTTCCGATCTNNNNNNGTCGGTAAAACTCGTGCCAGC‐3′, reverse: 5′‐GTGACTGGAGTTCAGACGTGTGCTCTTCCGATCTNNNNNNCATAGTGGGGTATCTAATCCCAGTTTG‐3′). The six random bases were used to enhance cluster separation on the flow cells during initial base call calibrations on the MiSeq platform. The first PCR was carried out with a 12 μl reaction volume containing 6.0 μl of 2 × KAPA HiFi HotStart ReadyMix (KAPA, Biosystems), 0.36 μl of each primer (10 μM), 4.28 μl of sterilized distilled H_2_O, and 1.0 μl of the template. The final concentration of each primer in this reaction mixture was 0.3 μM. The thermal cycle profile after an initial 3 min denaturation at 95°C was as follows (40 cycles): denaturation at 98°C for 20 s, annealing at 65°C for 15 s, and extension at 72°C for 15 s, with a final extension at 72°C for 5 min. The first PCRs were performed using four replicates to mitigate false negatives (PCR dropouts). In order to monitor contamination during the PCR process, blank samples were included. During the first PCR, PCR blanks (1 blank for every first PCR trails, 18 blanks in total) with 1.0 μl Milli‐Q water instead of template DNA were added. Thereafter, individual first PCR replicates were pooled and purified using SPRIselect Reagent Kit (Beckman Coulter), according to the manufacturer's instructions. Initially, the same volume of SPRI beads was added to each sample. The purified first PCR products were used as templates for the second PCR.

In the second PCR, the Illumina sequencing adapters and the 8 bp identifier indices were added using forward and reverse fusion primers (forward: 5′‐AATGATACGGCGACCACCGAGATCTACAXXXXXXXXACACTCTTTCCCTACACGACGCTCTTCCCATCT‐3′, reverse: 5′‐CAAGCAGAAGACGGCATACGAGATXXXXXXXXGTGACTGGAGTTCAGACGTGTGCTCTTCCGATCT‐3′). The eight X bases represent dual‐index sequences inserted to identify different samples. The second PCR was carried out with a 12 μl reaction volume containing 6.0 μl × KAPA HiFi HotStart ReadyMix, 2 μl of each primer (1.8 μM), 1.0 μl of sterilized distilled H_2_O, and 1.0 μl of template. The final concentration of each primer in this reaction mixture was 0.3 μM. The thermal cycle profile after an initial 3 min denaturation at 95°C was as follows (12 cycles): denaturation at 98°C for 20 s; combined annealing and extension at 72°C (shuttle PCR) for 20 s, with a final extension at 72°C for 5 min. The indexed second PCR products were pooled (i.e., one pooled second PCR product that included all samples).

The pooled libraries were loaded on a 2% E‐Gel SizeSelect (Thermo Fisher Scientific), and the target size (approximately 370 bp) was collected. The DNA size distribution of the library was estimated using an Agilent 2100 BioAnalyzer (Agilent), and the library concentration was quantified using a Qubit dsDNA HS assay kit and a Qubit 3.0 (Thermo Fisher Scientific). The amplicon libraries were sequenced on the MiSeq platform at Ryukoku University using a MiSeq v2 Regent Kit for 2 × 150 bp pair‐end (Illumina) according to the manufacturer's protocol.

### 2.5 Bioinformatic analysis and contamination controls

All data preprocessing and analyses of MiSeq raw reads were performed using USEARCH v10.0.240 (Edgar, 2010) as follows: (a) Paired‐end reads (forward and reverse reads) were merged using the “fastq_mergepairs” command with a default setting. During this process, too short reads (<100 bp) after tail trimming and the paired reads with too many differences (>5 positions) in the aligned region (c. 65 bp) were discarded; (b) primer sequences were removed from the merged reads using the “fastx_truncate” command; (c) the reads without primer sequences were then filtered using the “fastq_filter” command to remove low‐quality reads with an expected error rate (Edgar & Flyvbjerg, 2015) of >1% and short reads of <100 bp; (d) the preprocessed reads were dereplicated using the “fastx_uniques” command, and all singletons, doubletons, and tripletons were removed from the subsequent analysis following the recommendation by Edgar (2010); (e) the dereplicated reads were denoised using the “unoise3” command to generate amplicon sequence variants (ASVs) and remove all putatively chimeric, erroneous sequences and partial ASVs with less than 10 reads; and (f) finally, ASVs were subjected to taxonomic assignments to species names using the “usearch_global” command with a sequence identity of >98.5% (two nucleotide differences allowed) with the reference sequences and a query coverage of ≥90%.

After the taxonomic assignments, some modifications had to be made since (a) some closely related species could not be distinguished using the amplified region of 12S rRNA gene, and (b) the program returned species that were unlikely to inhabit the study areas (e.g., marine species) or taxonomic groups other than fish (e.g., mammals). In these two scenarios, the taxonomic assignment was modified as follows: In the first scenario, some closely related species were merged and assigned to the genus, but if there was only one species from the taxa around study sites, it was reassigned to the single species. The distribution range of detected species was determined based on the results of conventional field surveys (the National Census on River and Dam Environments) and existing literature (Miyadi, Kawanabe, & Mizuno, 1976). In the second scenario, those species were removed from the data. In addition, according to Miya et al. (2015), MiFish‐U primers amplify teleost fish DNA. Therefore, cartilaginous fish were excluded from the data and only teleost fish data were used in this study.

To remove possible contaminants that were detected from the negative control samples, we subtracted contaminant reads from field samples as follows (Nguyen, Smith, Peay, & Kennedy, 2015; Port et al., 2016): Read counts obtained from field blanks and extraction blanks were subtracted from each field sample processed on the same day, and read counts obtained from PCR blanks were subtracted from each field sample included in the PCR run.

### 2.6 National census data

To confirm the fish species detected in this study, we examined previous survey data based on direct capture and observation methods in these dam reservoirs. In Japan, the Ministry of Land, Infrastructure and Transport carries out National Census on River and Dam Environments, in which capture surveys are conducted using a cast net, a gill net, and a stationary net. In principle, these surveys are carried out once in every 5 years, once in the summer, and once in the autumn of the survey year. In this study, using the data obtained from the National Census on River and Dam Environments, we evaluated the effectiveness of eDNA metabarcoding method in the dam reservoirs. Specifically, we used data obtained from the national census conducted in 1999, 2004, 2009, and 2014 at the Miharu dam reservoir, and in 1999, 2006, 2011, and 2015 at the Okawa dam reservoir. Unfortunately, no such national census has been conducted in Sugo dam reservoir (River Environmental Database, http://www.nilim.go.jp/lab/fbg/ksnkankyo/), and no comparisons were made using conventional methods.

### 2.7 Statistical analyses

We used the function “rarecurve” in the vegan package of R version 3.6.0 (R Core Team, 2019) to confirm whether the sequencing depth was sufficient to detect the α‐diversity in each sample (results in Appendix S2). After that, we converted the read numbers to presence/absence data and used it as binary data for all statistical analyses.

In each dam reservoir, a generalized additive model (GAM) with a Poisson distribution was used to evaluate the monthly differences in the number of species detected by eDNA metabarcoding. In this model, the sampling month was set as a smoothing term and the average number of detected species as a response variable. We used the “gam” function in mgcv package of R version 3.6.0 for this analysis.

The differences in fish community compositions were visualized using the two‐dimensional nonmetric multidimensional scaling (NMDS) with 100 separate runs of real data. For NMDS, the community dissimilarity was calculated based on incidence‐based Jaccard indices. The differences in the fish community structures between sampling seasons and locations were evaluated using a permutational multivariate analysis of variance (PERMANOVA). For PERMANOVA, we used Jaccard similarity matrix and calculated the statistical values with 999 permutations. We also tested heterogeneity of dispersion between sources using a permutational analysis of multivariate dispersion (PERMDISP) to determine whether the significant values in PERMANOVA were due to different multivariate means or different heterogeneity of the groups. We used “metaMDS” function for NMDS ordination, “adonis” function for PERMANOVA, and “betadisper” and “permutest” functions for PERMDISP in vegan package. In these analyses, the sampling seasons were defined as spring (March–May), summer (June–August), autumn (September–November), and winter (December–February).

In addition, Wilcoxon rank sum test was conducted on the two groups (shore and offshore), in each dam reservoir, in order to investigate whether there is a difference in the number of detected species between shore and offshore samples. We used the “Wilcox.exact” functions, in exactRankTests package of R version 3.6.0, for Wilcoxon rank sum test.

## 3 RESULTS

### 3.1 MiSeq sequencing and taxonomic assignment

In the Miharu dam reservoir, the MiSeq paired‐end sequencing of the 235 PCR libraries (including 210 field samples and 25 negative controls) yielded a total of 5,303,849 raw reads. In the Okawa dam reservoir, 63 PCR libraries (including 54 field samples and nine negative controls) yielded a total of 5,048,500 raw reads. In the Sugo dam reservoir, 80 PCR libraries (including 60 field samples and 20 negative controls) yielded a total of 3,729,915 raw reads. These raw reads were then merged, quality‐filtered, and denoised, resulting in a total of 3,697,029 reads, 3,753,568 reads, and 3,006,143 reads in Miharu, Okawa, and Sugo dam reservoir, respectively. Finally, some modifications were made after taxonomic assignments which resulted in a total of 3,412,177 reads, 3,383,939 reads, and 2,680,733 reads from the Miharu, Okawa, and Sugo dam reservoirs, respectively (Appendix S3 for details). These processed reads were used for species identification (Table S1). Based on bioinformatics analysis, we detected 21, 24, and 22 species in Miharu, Okawa, and Sugo dam reservoirs, respectively (Appendix S4). In this dataset, we considered *Carassius auratus* subspecies group and *Carassius* *gibelio* as *Carassius* spp., *Hemibarbus barbus* and *Hemibarbus labeo* as *Hemibarbus* spp., *Salvelinus leucomaenis* and *S. leucomaenis pluvius* as *Salvelinus leucomaenis*, and all *Rhinogobius*, except for *Rhinogobius flumineus*, as *Rhinogobius* spp., due to their high DNA‐sequence similarities (<2 bp differences), and also because these species were treated and evaluated in the same way, in the conventional surveys.

### 3.2 Comparison of conventional survey and eDNA metabarcoding data

**FIGURE 2:**
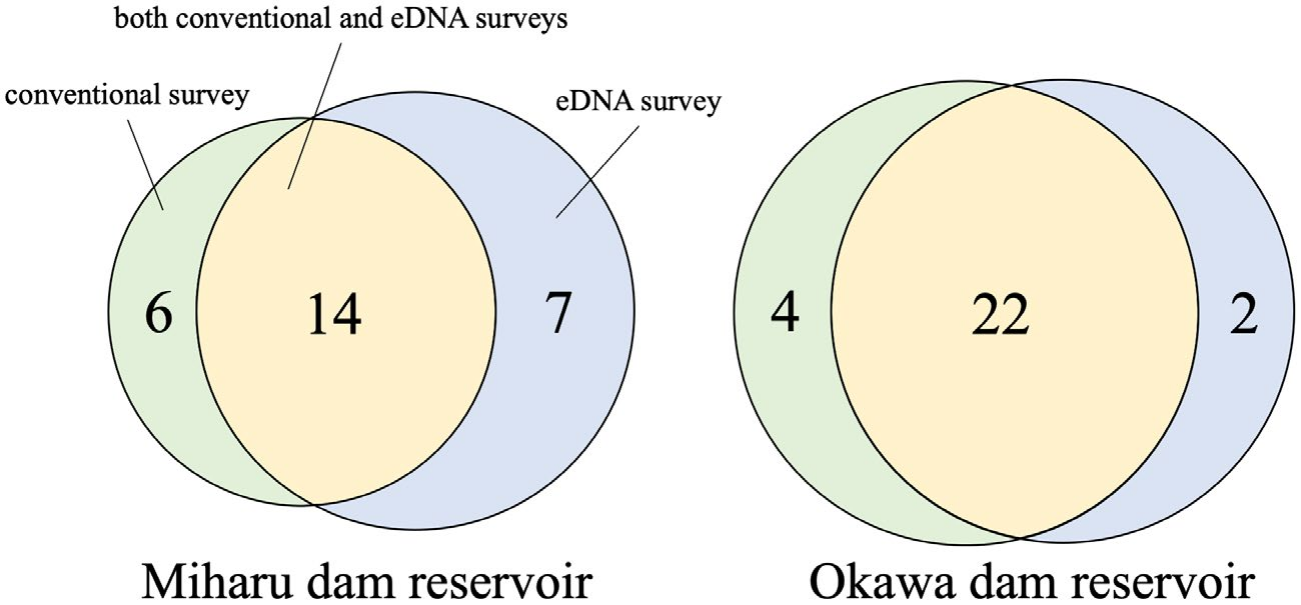
Venn diagram showing the number of species detected in conventional and eDNA surveys. Green, yellow, and blue denote the number of detected species only in conventional survey, the number of detected species both in conventional and eDNA surveys, and the number of detected species only in eDNA survey, respectively

In Miharu and Okawa dam reservoirs, a total of 27 and 28 species, respectively, were detected using either conventional or eDNA surveys (Appendix S5; Figure 2). Among them, in Miharu dam reservoir, 14 species were detected by both conventional and eDNA metabarcoding surveys, six species (*Rhodeus ocellatus ocellatus*, *Misgurnus dabryanus*, *Cobitis biwae*, *Lefua echigonia*, *Tachysurus tokiensis*, and *Oncorhynchus masou*) were detected only by the conventional survey, and seven species (*Ctenopharyngodon idella*, *Tribolodon sachalinensis*, *Silurus asotus*, *Oncorhynchus keta*, *Micropterus dolomieu*, *Tridentiger* sp., and *Channa argus*) were detected only by the eDNA metabarcoding method. In Okawa dam reservoir, 22 species were detected by both conventional and eDNA surveys, four species (*Opsariichthys uncirostris*, *C. biwae*, *S. asotus*, and *M. dolomieu*) were detected only by the conventional survey, and two species (*Lepomis macrochirus* and *Micropterus salmoides*) were detected only by the eDNA metabarcoding method. In Sugo dam reservoir, conventional survey was not conducted, so the results obtained from eDNA metabarcoding method could not be compared with the results of the conventional survey. The eDNA metabarcoding detected 70% and 84% of the species identified by the long‐term conventional surveys in Miharu and Okawa dam reservoirs, respectively. The eDNA metabarcoding method detected 83% and 91% of the species that were identified by the conventional surveys in the Miharu and Okawa dam reservoirs, respectively.

### 3.3 Sampling season

**FIGURE 3:**
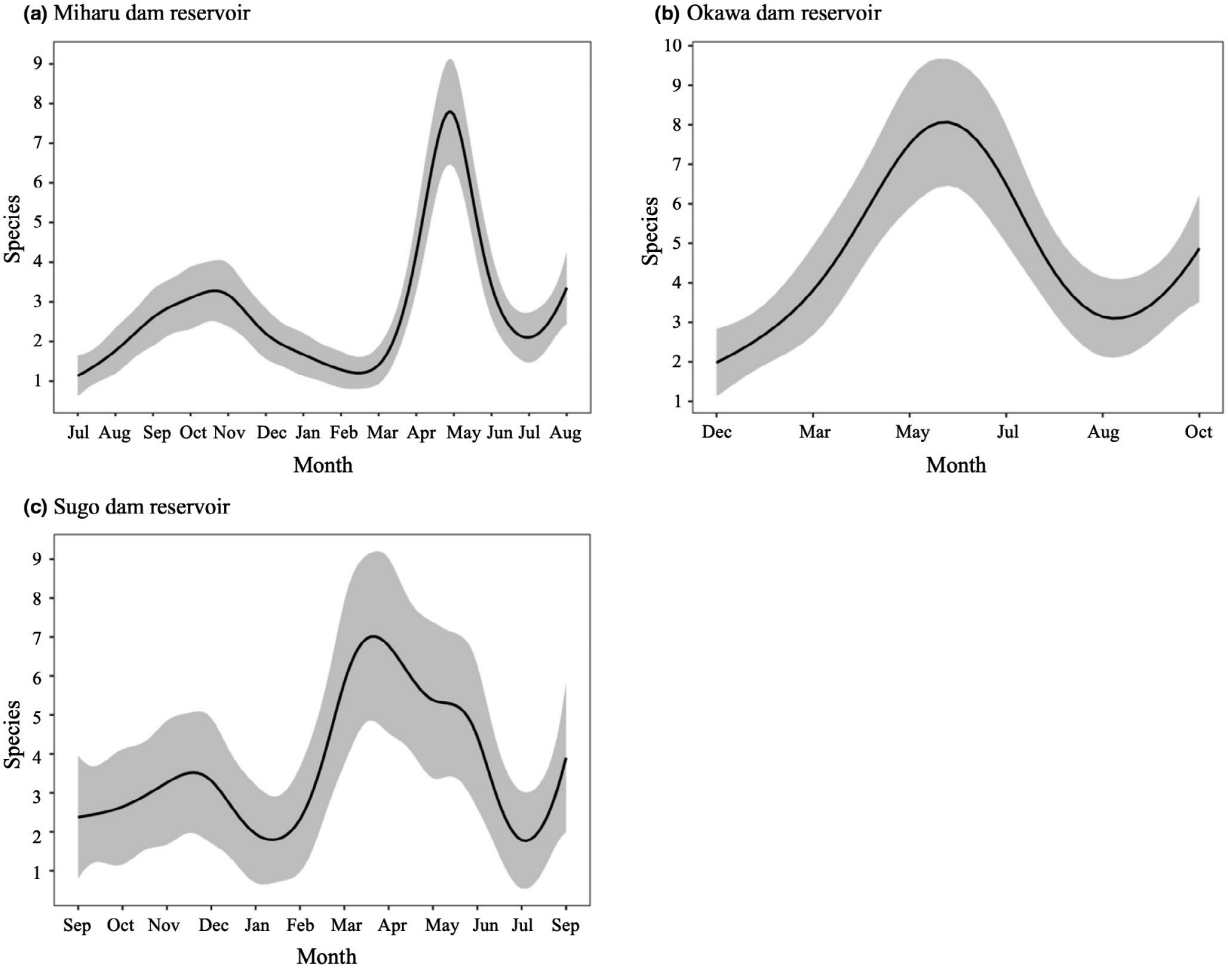
Smooth function of sampling month produced by the GAM for (a) Miharu dam reservoir, (b) Okawa dam reservoir, and (c) Sugo dam reservoir. The solid line represents the predicted value of the number of species detected as a function of sampling month. The glay shaded area represent 95% confidence interval. See the detail values in Table 2

The number of species detected in each dam reservoir using eDNA metabarcoding method varied significantly from month to month (All three dam reservoirs *p* < .01; Figure 3; Appendix S5). In Miharu dam reservoir, a large number of species were detected from April to May (Figure 3a), with the largest number of detected species recorded in May (Appendix S2a). In Okawa dam reservoir, a large number of species were detected from May to July (Figure 3b), with the largest number of detected species recorded in May (Appendix S2b). In Sugo dam reservoir, a large number of species were detected from March to June (Figure 3c), with the largest number of detected species recorded in March and June (Appendix S6c).

**FIGURE 4:**
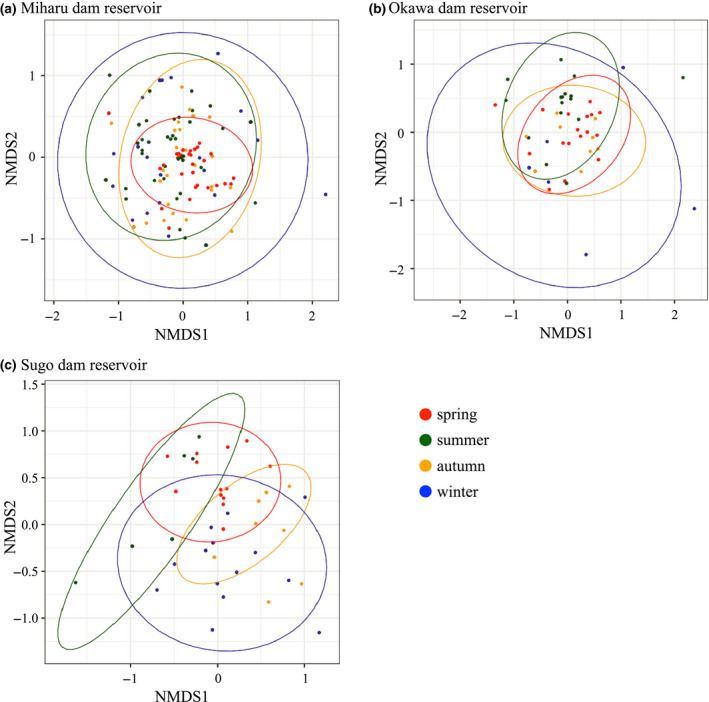
Nonmetric multidimensional scaling (NMDS) ordination of fish community compositions of all eDNA samples in each dam reservoir colored for seasons for (a) Miharu dam reservoir, (b) Okawa dam reservoir, and (c) Sugo dam reservoir. Fish community compositions were evaluated between sampling seasons. Spring—March to May (red); summer—June to August (green); autumn—September to November (orange); and winter—December to February (blue). The ellipses show the 95% confidence level based on the centroid calculated for each season

The NMDS results showed that species variation was different between sampling seasons (Figure 4). Specifically, there were few species variations between sampling sites in spring, and there was a large species variation between sampling sites in winter. This result was especially remarkable in the Miharu and Okawa dam reservoirs (Figure 4a,b). Moreover, the PERMANOVA analysis suggested that the community structure was significantly different between sampling seasons in all three dam reservoirs (All three dam reservoirs *p* < .001; Table 3). The subsequent PERMDISP analysis further indicated that the fish community structures were significantly heterogeneous during the sampling seasons in the Miharu dam reservoir (*p* < .001; Table 4a), whereas, in the Okawa and Sugo dam reservoirs, heterogeneity of the fish community structures was not significant (*p* = .459, .347; Table 4b, c).

### 3.4 Sampling location

**FIGURE 5:**
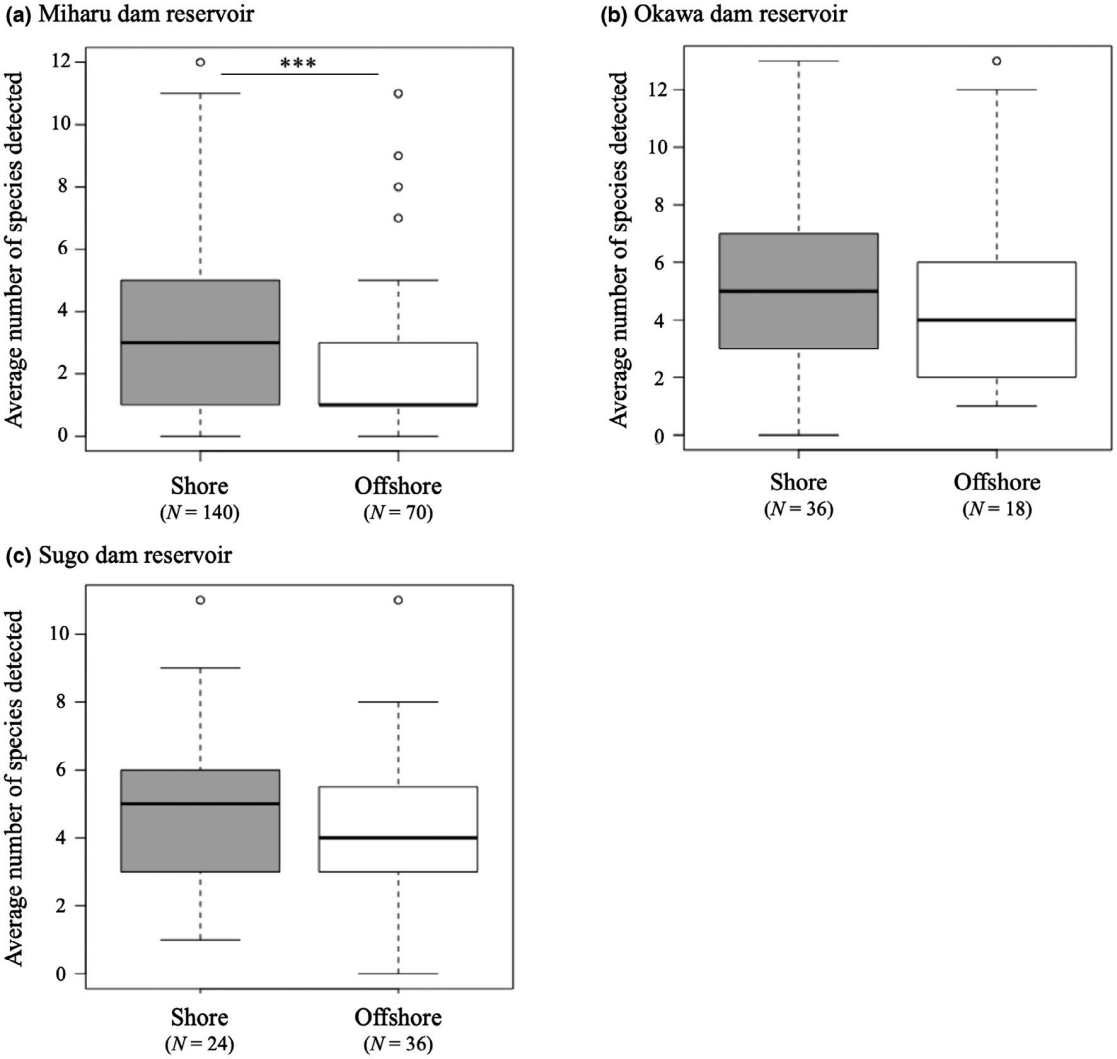
Comparison of the average number of species detected in shore and offshore for (a) Miharu dam reservoir, (b) Okawa dam reservoir, and (c) Sugo dam reservoir. *p* < .05, *p* < .01, and ***p < .001

**FIGURE 6:**
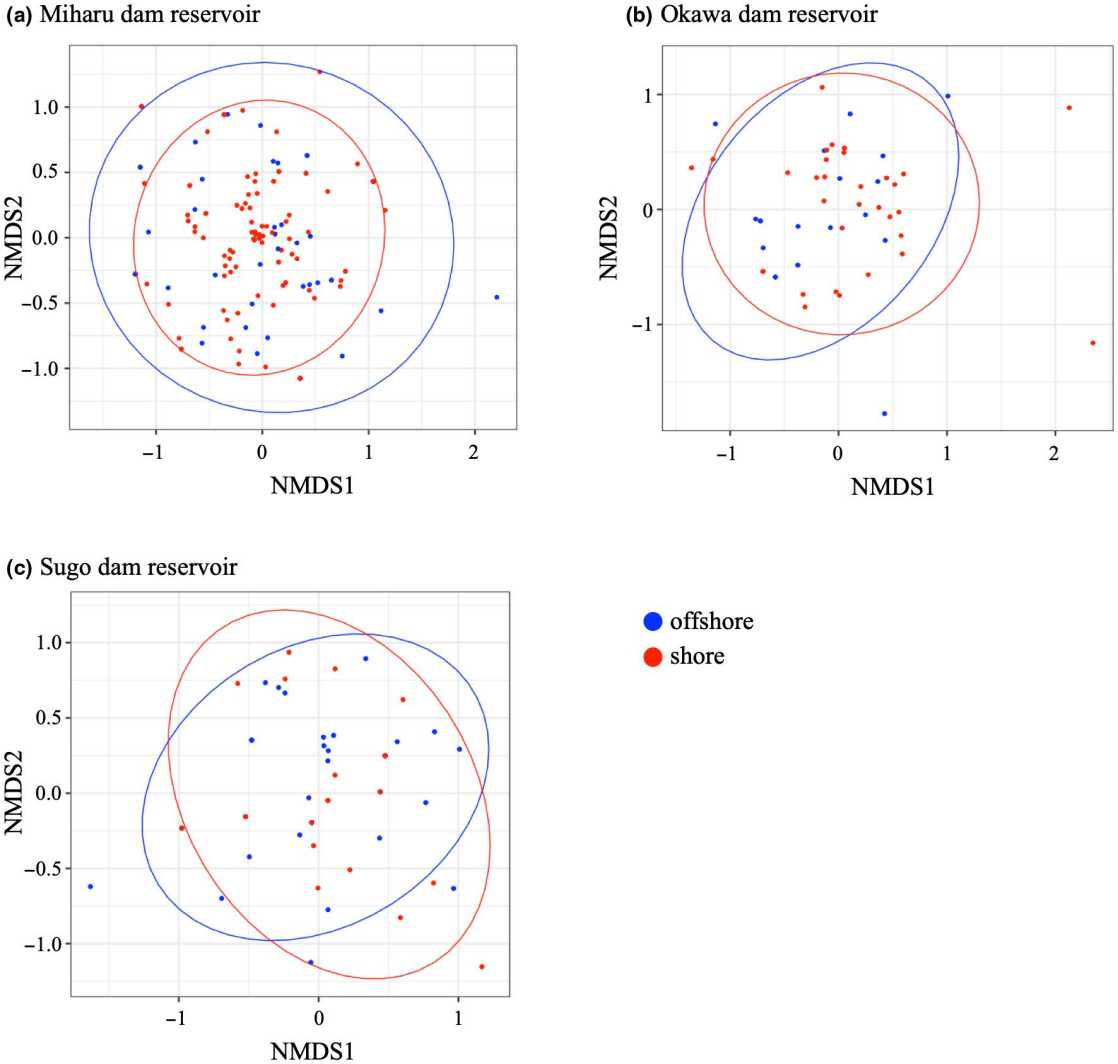
Nonmetric multidimensional scaling (NMDS) ordination of fish community compositions of all eDNA samples in each dam reservoir colored for location type for (a) Miharu dam reservoir, (b) Okawa dam reservoir, and (c) Sugo dam reservoir. Red and blue represent shore and offshore samples, respectively. The ellipses show the 95% confidence level based on the centroid calculated for each location type (shore or offshore)

By comparing the number of detected species in offshore and shore locations in each dam reservoir, we found that, in the Miharu dam reservoir, the number of species detected in shore was significantly greater than in offshore (*p* < .001; Figure 5a). In Okawa and Sugo dam reservoirs, the number of detected species was not significantly different between offshore and shore (*p* = .5028, .7824; Figure 5b,c). The results of NMDS showed that there was no substantial variation between sampling locations in all three dam reservoirs (Figure 6). However, the PERMANOVA analysis suggested that the community structure was significantly different between sampling locations in the Miharu dam reservoirs (*p* = .033; Table 3a), whereas, in Okawa and Sugo dam reservoirs, the community structure did not differ significantly between sampling locations (*p* = .753, .700; Table 3b,c). The subsequent PERMDISP analysis further indicated that fish community structures were significantly heterogeneous between the sampling locations in the Miharu dam reservoir (*p* = .02; Table 4a). In the Okawa and Sugo dam reservoirs however, heterogeneity of the fish community structures was not significant (*p* = .619, .74; Table 4b,c).

## 4 DISCUSSION

In this study, we found that the number of species detected by eDNA metabarcoding method differs, depending on sampling seasons. The number of detected species can be maximized by conducting the eDNA surveys in spring to early summer (i.e., the breeding seasons of the fish that inhabit there) in all three reservoirs. Our data provide an important insight into the optimal conditions of eDNA sampling, to detect the maximum number of species with minimum effort, by collecting water samples during the breeding season of the inhabiting fish.

Through eDNA metabarcoding, we detected 70% and 84% of the species identified by the previous long‐term surveys, conducted in the past 15 years, in Miharu and Okawa dam reservoirs, respectively. As shown in previous studies, our data also confirm that eDNA metabarcoding method is effective in evaluating entire fish communities (e.g., Fujii et al., 2019; Port et al., 2016; Shaw et al., 2016; Yamamoto et al., 2017). In addition, some species that were not identified in the previous surveys were detected by eDNA metabarcoding. In the Miharu dam reservoir, seven species (*C* *. idella*, *T. sachalinensis*, *S. asotus*, *O. keta*, *M. dolomieu*, *Tridentiger* sp., and *C. argus*) were detected only by eDNA metabarcoding. Among these, *S. asotus* and *Tridentiger* sp. have been captured by a large fixed net survey, and *C. idella* released in 2008 was visually confirmed in 2017, and also captured in 2019 by one of the authors (J. O.). However, these species were not detected by the national surveys. In addition, *C. argus* was also confirmed in 2019 by the same author. When these species were added to the species detected in previous surveys, we could detect 75% of the species detected in previous surveys through eDNA metabarcoding. Also, *M. dolomieu* are known to be distributed downstream of the reservoir according to previous surveys. However, for *T. sachalinensis*, *O. keta*, and *M. dolomieu*, the possibility of false positives was considered because the presence of these species has never been confirmed in the reservoir. Such spurious eDNA identifications may be the result of transportation of eDNA by secondary vectors, such as predator feces, boats, sewage inflow, and water currents (Deiner & Altermatt, 2014; Merkes, McCalla, Jensen, Gaikowski, & Amberg, 2014). In particular, the national census has confirmed the presence of a large number of cormorants (*Phalacrocorax carbo*) as potential source of predator feces in Miharu dam reservoir, so there is a high possibility of false positives due to the feces of the cormorants. In Okawa dam reservoir, the two species (*L. macrochirus* and *M. salmoides*) were detected only by eDNA metabarcoding. The presence of these species has never been reported previously. These two species are known to be distributed downstream of the dam reservoir according to the previous surveys. In addition, the Ouchi dam reservoir, which is located about 3 km west of the Okawa dam reservoir, serves as a fishing spot for *M. salmoides*, and there are *L. macrochirus* and *M. salmoides* in Lake Inawashiro, which is located only 20 km to its east. Therefore, these two species are considered possible false positives, for the reasons described above.

On the other hand, some species identified in previous survey were not detected by eDNA metabarcoding method. In Miharu dam reservoir, six species (*R. ocellatus ocellatus*, *M. dabryanus*, *C. biwae*, *L. echigonia*, *T. tokiensis*, and *Oncorhynchus masou masou*) were not detected, and as for *C. biwae*, *L. echigonia*, *T. tokiensis*, and *O. masou masou*, only one or two individuals were confirmed in previous 15 years of surveys. Therefore, it can be considered that these species were not detected because the amount of eDNA in the water sample was limited due to the small number of individuals present (Doi et al., 2017; Takahara et al., 2012). As for *R. ocellatus ocellatus* and *M. dabryanus*, in the previous surveys, most individuals were identified in a fore reservoir where the water sampling was not conducted in this study, and therefore, they may not have been detected. In the Okawa dam reservoir, four species (*O. uncirostris*, *C. biwae*, *S. asotus*, and *M. dolomieu*) were not detected. *O. uncirostris* has not been confirmed since the previous surveys in 2006, and it may not have been present when we collected the water samples. As for *M. dolomieu*, it is probable that they could not be detected because only two individuals were confirmed in previous surveys in 2011. Similarly, *S. asotus* could not be detected probably because only two individuals were confirmed in previous 15 years of surveys. *C. biwae* on the other hand may be difficult to detect from eDNA by surface water sampling because this species inhabits the sand beds or the bottom layer of a lake. In this study, water was only collected from the surface, but this problem could be solved by collecting water also in the vertical direction, as done in some previous studies (Hinlo et al., 2017; Moyer, Diaz‐Ferguson, Hill, & Shea, 2014; Wu et al., 2019).

As for the sampling seasons, the number of detected species increased from spring to early summer in all three dam reservoirs (Figure 3; Table 2). The numbers peaked in May in the Miharu and Okawa dam reservoirs, and in March and June in the Sugo dam reservoir (Appendix S2). The breeding season of most of fish that inhabit these three reservoirs is considered to be from spring to early summer. In the Okawa dam reservoir, the number of detected species also increased in October (Appendix S2). This may be because there are many species that reach the breeding season in autumn, such as *Plecoglossus altivelis* and Salmonidae species (e.g., *S. leucomaenis*, *Oncorhynchus mykiss*, and *O. masou masou*), in this reservoir, compared to the other two reservoirs. Previous species‐specific studies focusing on target species have also reported that eDNA concentrations or the detection frequency increases during the breeding season (Bylemans et al., 2017; Fukumoto, Ushimaru, & Minamoto, 2015; Spear, Groves, Williams, & Waits, 2015). Therefore, even when eDNA metabarcoding is used, it is considered that eDNA is easier to capture in the breeding season, resulting in an increase in the number of detected species.

**TABLE 2:** Summary results of generalized additive models for seasonal differences of the number of species detected in (a) Miharu dam reservoir, (b) Okawa dam reservoir, and (c) Sugo dam reservoir

Approximate significance of smooth terms	*e.d.f.*	Ref. *df*	Chi. sq	*p*
(a) Miharu dam reservoir				
*s*(month)	9.888	11.51	184.6	<.001***
(b) Okawa dam reservoir				
*s*(month)	4.457	4.873	46.48	<.001***
(c) Sugo dam reservoir				
*s*(month)	8.709	10.03	33.67	<.001***

*** *p* < .001.

Furthermore, in the Miharu dam reservoir, species variations between sampling seasons were small in spring and large in winter (Table 4; Figure 4). In spring, which is the breeding season, it is considered that not only a large number of species can be detected but also the representative species that live in the dam reservoir can be detected at all locations. On the contrary, because there was a large variation in winter, it is difficult to detect representative species in each sampling location. Previous studies have also reported that detection probability of eDNA is lower in winter, compared to spring (Buxton, Groombridge, & Griffiths, 2018; Turner et al., 2014). Therefore, it can be said that the same tendency was observed in this study. In Okawa and Sugo dam reservoirs, the fish community structures were significantly different between sampling seasons. Therefore, the eDNA survey should probably be conducted in multiple seasons, including spring, rather than only in spring, at some types of reservoirs.

As for the sampling locations, in the Miharu dam reservoir, it was shown that the number of detected species increased by sampling water at shore locations (Figure 5a). On the contrary, in Okawa and Sugo reservoirs, there was no difference in the numbers of detected species at offshore and shore locations (Figure 5b,c).

Furthermore, in the Miharu dam reservoir, the species variations between sampling locations were small in shore and large offshore (Table 4; Figure 6). Therefore, it can be considered that not only large number of species can be detected on the shore, but also representative species that live in the dam reservoir can be detected. On the other hand, in Okawa and Sugo dam reservoir, there was no difference in species composition between sampling locations (Table 3), nor were there differences in species variation (Table 4). However, in the Okawa dam reservoir, appearance frequency tends to be higher on the shore compared to offshore locations (Table S1). Furthermore, the Sugo dam reservoir is small compared to the other two dam reservoirs (Table 1), and the distances between sampling sites are relatively short (Figure 1). As a result, the difference between shore and offshore locations cannot be observed, due to the short distances between sites. From the results of this study, it can be said that it is possible to detect most species even with water sampling only from shore locations. Hänfling et al. (2016) suggested that eDNA could accumulate in the shore locations and that shore sampling may be suitable for detection of most species. Shore sampling can be conducted with less effort compared to offshore sampling because the water can be accessed directly without special equipment. However, according to Lawson Handley et al. (2019), there is no difference in the number of detected species in water samples collected offshore and shore locations in winter, but there is a clear difference in summer. Detailed research on the water sampling locations is still needed.

**TABLE 3:** Summary of PERMANOVA results for sampling season and location differences in fish community composition for each dam reservoir

	*df*	Sum of Sqs	Mean Sqs	*F*	*R* ^2^	*p*
(a) Miharu dam reservoir						
Season	3	3.183	1.06099	3.3575	0.05854	<.001***
Location	1	0.627	0.62738	1.9854	0.01154	.033*
Residuals	160	50.560	0.31600		0.92992	
Total	164	54.371			1.00000	
(b) Okawa dam reservoir						
Season	3	2.1927	0.73091	2.5574	0.13611	<.001***
Location	1	0.1984	0.19841	0.6942	0.01232	.753
Residuals	48	13.7183	0.28580		0.85157	
Total	52	16.1094			1.00000	
(c) Sugo dam reservoir						
Season	3	2.9258	0.97527	6.7177	0.26943	<.001***
Location	1	0.0937	0.09370	0.6454	0.00863	.700
Residuals	54	7.8396	0.14518		0.72194	
Total	58	10.8591			1.00000	

* *p* < .05.

*** *p* < .001.

**TABLE 4:** Summary of PERMDISP results for inter‐season and inter‐location heterogeneity of samples in each dam reservoir

	*df*	Sum of Sqs	Mean Sqs	*F*	*p*
(a) Miharu dam reservoir					
Season	3	0.40916	0.136387	10.192	<.001***
Residuals	161	2.15446	0.013382		
Location	1	0.09057	0.090572	6.3252	.02*
Residuals	164	2.33405	0.014319		
(b) Okawa dam reservoir					
Season	3	0.0690	0.022999	0.8688	.459
Residuals	49	1.2971	0.026471		
Location	1	0.00354	0.003543	0.2574	.619
Residuals	51	0.70203	0.013765		
(c) Sugo dam reservoir					
Season	3	0.09679	0.032262	1.1242	.347
Residuals	55	1.57837	0.028698		
Location	1	0.00258	0.002580	0.1057	.74
Residuals	57	1.39174	0.024417		

* *p* < .05.

*** *p* < .001.

From the results of both sampling seasons and locations, it can be concluded that the number of species detected by eDNA may be maximized by collecting water in shore locations during the breeding season. This is because fish tend to gather towards the shore, where the spawning substrate is present during the breeding season, even if the species do not normally inhabit the shore, and the amount of eDNA released is also increased. In fact, it has been reported that *M. salmoides*, an exotic fish that was detected in all three dam reservoirs, laid eggs along the shore locations from May to June and hatched juveniles gathered on the shore of the reservoir (Ministry of the Environment, 2004; Nakai, 2004).

In this study, we evaluated the effect of sampling seasons and locations in eDNA metabarcoding. By doing so, we have shown that maximum number of species can be detected with minimum effort, by collecting water from shore locations during the breeding seasons of the fish that inhabit there. These results could contribute immensely in the determination of sampling seasons and locations on fish fauna survey in the future.

## CONFLICT OF INTEREST

The authors declare that there is no conflict of interest regarding the publication of this article.

## AUTHOR CONTRIBUTION

**Kana Hayami:** Data curation (equal); Investigation (equal); Software (equal); Visualization (lead); Writing‐original draft (equal); Writing‐review & editing (equal). **Masayuki K. Sakata:** Data curation (equal); Investigation (equal); Software (equal); Visualization (supporting); Writing‐review & editing (equal). **Takashi Inagawa:** Data curation (supporting); Investigation (equal); Writing‐review & editing (equal). **Jiro Okitsu:** Conceptualization (supporting); Data curation (supporting); Investigation (equal); Writing‐review & editing (supporting). **Izumi Katano:** Data curation (supporting); Investigation (equal); Writing‐review & editing (equal). **Hideyuki Doi:** Conceptualization (supporting); Data curation (supporting); Investigation (supporting); Writing‐review & editing (equal). **Katsuki Nakai:** Conceptualization (supporting); Data curation (supporting); Writing‐review & editing (equal). **Hidetaka Ichiyanagi:** Conceptualization (supporting); Funding acquisition (equal); Investigation (supporting); Writing‐review & editing (equal). **Ryo O. Gotoh:** Software (supporting); Writing‐review & editing (equal). **Masaki Miya:** Software (supporting); Writing‐review & editing (equal). **Hirotoshi Sato:** Investigation (equal); Writing‐review & editing (equal). **Hiroki Yamanaka:** Investigation (equal); Writing‐review & editing (equal). **Toshifumi Minamoto:** Conceptualization (lead); Data curation (supporting); Funding acquisition (lead); Investigation (supporting); Project administration (lead); Supervision (lead); Writing‐original draft (equal); Writing‐review & editing (equal).

## Supporting information

Appendix S1‐S6

## Data Availability

The Table S1 and the raw data are deposited to Dryad (https://doi.org/10.5061/dryad.zkh189374).
