# Supplementary material for: Effects of sampling seasons and locations on fish environmental DNA metabarcoding in dam reservoirs
Source: Ecol Evol. 2020 May 6;10(12):5354–67. doi: 10.1002/ece3.6279 (PMC7319172; doi:10.1002/ece3.6279)

Appendix 1

(a) Miharu dam reservoir

| Sampling site ID | Sampling location | Latitude | Longtitude |
| --- | --- | --- | --- |
| M1 | shore | 37.41097 | 140.480207 |
| M2 | offshore | 37.410941 | 140.480921 |
| M3 | shore | 37.411149 | 140.482167 |
| M4 | shore | 37.40873 | 140.486011 |
| M5 | offshore | 37.407912 | 140.486304 |
| M6 | shore | 37.407332 | 140.486611 |
| M7 | shore | 37.403618 | 140.486424 |
| M8 | offshore | 37.4036 | 140.485676 |
| M9 | shore | 37.403486 | 140.484612 |
| M10 | shore | 37.407289 | 140.478546 |
| M11 | offshore | 37.406428 | 140.47862 |
| M12 | shore | 37.405563 | 140.478379 |
| M13 | offshore | 37.40719 | 140.481009 |
| M14 | shore | 37.393733 | 140.502589 |
| M15 | shore | 37.415968 | 140.485558 |

(b) Okawa dam reservoir

| Sampling site ID | Sampling location | Latitude | Longtitude |
| --- | --- | --- | --- |
| O1 | shore | 37.340168 | 139.912421 |
| O2 | offshore | 37.339994 | 139.911501 |
| O3 | shore | 37.339262 | 139.909836 |
| O4 | shore | 37.336996 | 139.915887 |
| O5 | offshore | 37.336361 | 139.9148 |
| O6 | shore | 37.336424 | 139.911917 |
| O7 | shore | 37.331475 | 139.918944 |
| O8 | offshore | 37.330547 | 139.918146 |
| O9 | shore | 37.32948 | 139.917229 |

(c) Sugo dam reservoir

| Sampling site ID | Sampling location | Latitude | Longtitude |
| --- | --- | --- | --- |
| S1 | shore | 35.004258 | 134.635436 |
| S2 | offshore | 35.004293 | 134.635281 |
| S3 | offshore | 35.004302 | 134.635143 |
| S4 | offshore | 35.004323 | 134.634958 |
| S5 | shore | 35.004351 | 134.634762 |

Appendix 2 (1)

Appendix 2 (2)

Appendix 2(3)

Appendix 2(4)

Appendix 3

|  | **Raw read** | | **Merged** | | **Quality filter** | | **Denoise** | | **Final** | |
| --- | --- | --- | --- | --- | --- | --- | --- | --- | --- | --- |
| **Study site** | Field | NC | Field | NC | Field | NC | Field | NC | Field | NC |
| **Miharu dam** | 5,192,305 | 111,544 | 4,397,061 | 85,226 | 4,355,414 | 82,176 | 3,626,588 | 70,441 | 3,378,647 | 33,530 |
| **Okawa dam** | 4,248,593 | 799,907 | 4,046,299 | 736,594 | 4,008,379 | 727,804 | 3,240,924 | 512,644 | 2,998,796 | 385,143 |
| **Sugo dam** | 3,044,902 | 685,013 | 2,979,226 | 662,039 | 2,963,507 | 609,777 | 2,450,922 | 555,221 | 2,137,087 | 543,646 |

Field means the reads of field samples, and NC means the reads of negative control samples.

Appendix 4

| Family | Species |
| --- | --- |
| Cyprinidae | *Cyprinus carpio* |
|  | *Carassius* spp. |
|  | *Carassius cuvieri* |
|  | *Opsariichthys platypus* |
|  | *Ctenopharyngodon idellus* |
|  | *Phoxinus lagowskii steindachneri* |
|  | *Tribolodon sachalinensis* |
|  | *Tribolodon hakonensis* |
|  | *Pseudorasbora parva* |
|  | *Gnathopogon elongatus* |
|  | *Hemibarbus barbus* |
| Cobitidae | *Misgurnus anguillicaudatus* |
| Siluridae | *Silurus asotus* |
| Salmonidae | *Oncorhynchus keta* |
| Centrarchidae | *Lepomis macrochirus macrochirus* |
|  | *Micropterus salmoides* |
|  | *Micropterus dolomieu* |
| Gobiidae | *Tridentiger* sp*.* |
|  | *Rhinogobius* spp*.* |
|  | *Gymnogobius urotaenia* |
| Channidae | *Channa argus* |

1. Miharu dam reservoir

(b) Okawa dam reservoir

| Family | Species |
| --- | --- |
| Cyprinidae | *Cyprinus carpio* |
|  | *Carassius* spp. |
|  | *Opsariichthys platypus* |
|  | *Candidia temminckii* |
|  | *Phoxinus lagowskii steindachneri* |
|  | *Tribolodon hakonensis* |
|  | *Pseudorasbora parva* |
|  | *Biwia zezera* |
|  | *Pseudogobio esocinus* |
|  | *Hemibarbus barbus* |
|  | *Squalidus chankaensis biwae* |
| Cobitidae | *Misgurnus anguillicaudatus* |
| Amblycipitidae | *Liobagrus reini* |
| Osmeridae | *Hypomesus nipponensis* |
|  | *Plecoglossus altivelis* |
| Salmonidae | *Salvelinus leucomaenis* |
|  | *Oncorhynchus mykiss* |
|  | *Oncorhynchus masou masou* |
| Centrarchidae | *Lepomis macrochirus macrochirus* |
|  | *Micropterus salmoides* |
|  | *Cottus pollux* |
| Gobiidae | *Tridentiger brevispinis* |
|  | *Rhinogobius* spp*.* |
|  | *Gymnogobius urotaenia* |

(c) Sugo dam reservoir

| Family | Species |
| --- | --- |
| Cyprinidae | *Cyprinus carpio* |
|  | *Carassius* spp. |
|  | *Carassius cuvieri* |
|  | *Opsariichthys platypus* |
|  | *Candidia temminckii* |
|  | *Candidia sieboldii* |
|  | *Phoxinus lagowskii steindachneri* |
|  | *Tribolodon hakonensis* |
|  | *Pseudorasbora parva* |
|  | *Biwia zezera* |
|  | *Pseudogobio esocinus* |
|  | *Hemibarbus* spp. |
|  | *Squalidus chankaensis biwae* |
| Cobitidae | *Misgurnus anguillicaudatus* |
|  | *Lefua* sp. 1 |
| Centrarchidae | *Lepomis macrochirus macrochirus* |
|  | *Micropterus salmoides* |
|  | *Cottus pollux* |
| Gobiidae | *Tridentiger* sp. |
|  | *Rhinogobius flumineus* |
|  | *Rhinogobius* spp*.* |
|  | *Gymnogobius urotaenia* |

Appendix 5

1. Miharu dam reservoir

| Species | Conventional survey | eDNA survey |
| --- | --- | --- |
| *Cyprinus carpio* | ◯ | ◯ |
| *Carassius* spp. | ◯ | ◯ |
| *Carassius cuvieri* | ◯ | ◯ |
| *Rhodeus ocellatus ocellatus* | ◯ |  |
| *Opsariichthys platypus* | ◯ | ◯ |
| *Ctenopharyngodon idellus* |  | ◯ |
| *Phoxinus lagowskii steindachneri* | ◯ | ◯ |
| *Tribolodon sachalinensis* |  | ◯ |
| *Tribolodon hakonensis* | ◯ | ◯ |
| *Pseudorasbora parva* | ◯ | ◯ |
| *Gnathopogon elongatus* | ◯ | ◯ |
| *Hemibarbus barbus* | ◯ | ◯ |
| *Misgurnus anguillicaudatus* | ◯ | ◯ |
| *Misgurnus dabryanus* | ◯ |  |
| *Cobitis biwae* | ◯ |  |
| *Lefua echigonia* | ◯ |  |
| *Tachysurus tokiensis* | ◯ |  |
| *Silurus asotus* |  | ◯ |
| *Oncorhynchus keta* |  | ◯ |
| *Oncorhynchus masou masou* | ◯ |  |
| *Lepomis macrochirus macrochirus* | ◯ | ◯ |
| *Micropterus salmoides* | ◯ | ◯ |
| *Micropterus dolomieu* |  | ◯ |
| *Tridentiger* sp*.* |  | ◯ |
| *Rhinogobius* spp*.* | ◯ | ◯ |
| *Gymnogobius urotaenia* | ◯ | ◯ |
| *Channa argus* |  | ◯ |

(b) Okawa dam reservoir

| Species | Conventional survey | eDNA survey |
| --- | --- | --- |
| *Cyprinus carpio* | ◯ | ◯ |
| *Carassius* spp. | ◯ | ◯ |
| *Opsariichthys uncirostris* | ◯ |  |
| *Opsariichthys platypus* | ◯ | ◯ |
| *Candidia temminckii* | ◯ | ◯ |
| *Phoxinus lagowskii steindachneri* | ◯ | ◯ |
| *Tribolodon hakonensis* | ◯ | ◯ |
| *Pseudorasbora parva* | ◯ | ◯ |
| *Biwia zezera* | ◯ | ◯ |
| *Pseudogobio esocinus* | ◯ | ◯ |
| *Hemibarbus barbus* | ◯ | ◯ |
| *Squalidus chankaensis biwae* | ◯ | ◯ |
| *Misgurnus anguillicaudatus* | ◯ | ◯ |
| *Cobitis biwae* | ◯ |  |
| *Silurus asotus* | ◯ |  |
| *Liobagrus reini* | ◯ | ◯ |
| *Hypomesus nipponensis* | ◯ | ◯ |
| *Plecoglossus altivelis* | ◯ | ◯ |
| *Salvelinus leucomaenis* | ◯ | ◯ |
| *Oncorhynchus mykiss* | ◯ | ◯ |
| *Oncorhynchus masou masou* | ◯ | ◯ |
| *Lepomis macrochirus macrochirus* |  | ◯ |
| *Micropterus salmoides* |  | ◯ |
| *Micropterus dolomieu* | ◯ |  |
| *Cottus pollux* | ◯ | ◯ |
| *Tridentiger brevispinis* | ◯ | ◯ |
| *Rhinogobius* spp*.* | ◯ | ◯ |
| *Gymnogobius urotaenia* | ◯ | ◯ |

Appendix 6


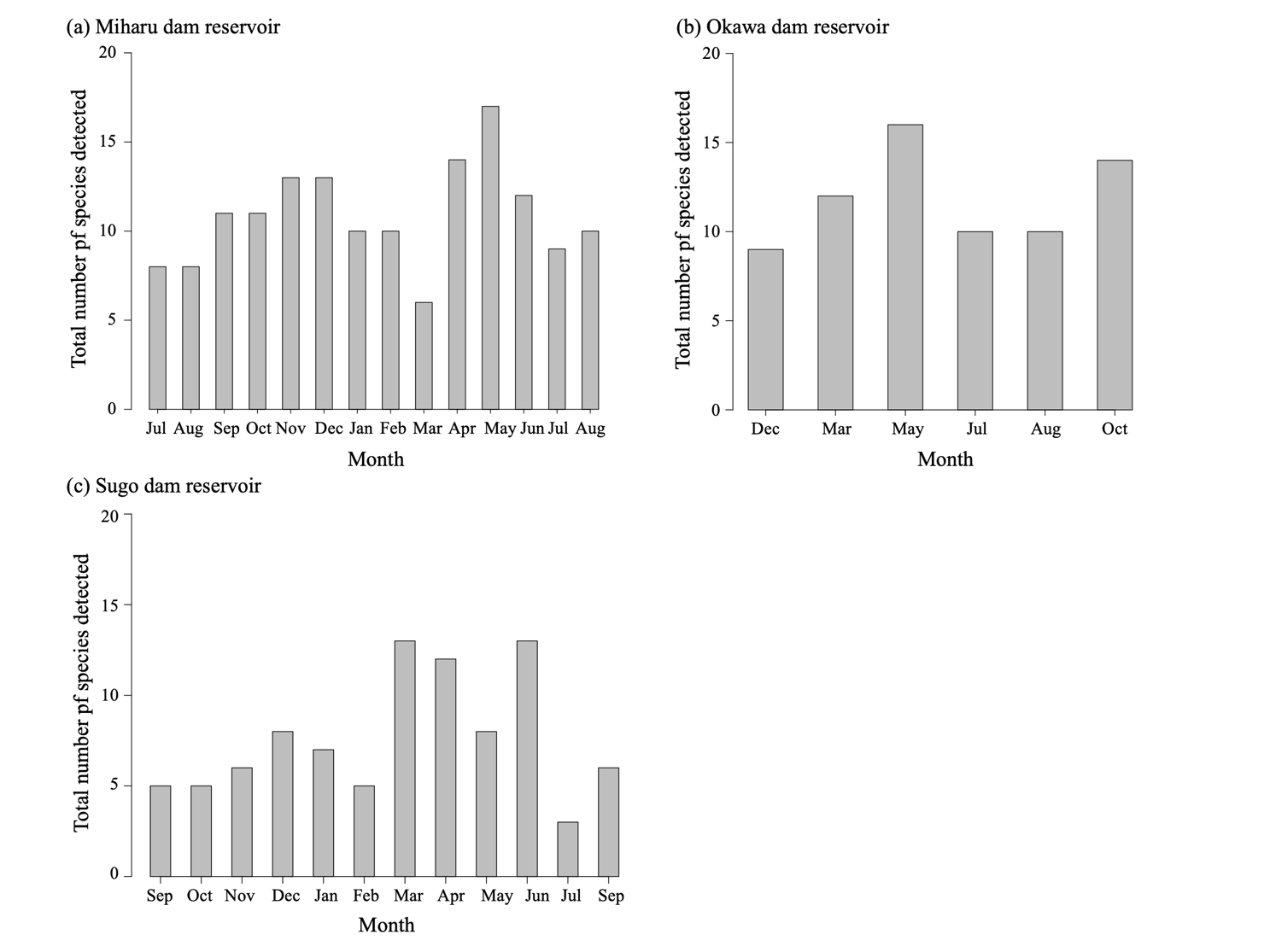

Supplement: Supplementary file 1 — Appendix S1‐S6 [file ECE3-10-5354-s001.docx]
